# Outcomes of Osteochondral Allograft Transplantation: A Comparative Study of BioUni and Snowman Techniques for Ovoid Lesions

**DOI:** 10.7759/cureus.46958

**Published:** 2023-10-13

**Authors:** Carlo Coladonato, Andres R Perez, Adeeb J Hanna, Michael P Campbell, Henson Destine, Azra N Dees, Emma E Johnson, Bradford S Tucker, Kevin B Freedman

**Affiliations:** 1 Sports Medicine, Rothman Orthopaedic Institute, Philadelphia, USA

**Keywords:** patient reported outcomes, oca, allograft, articular cartilage, knee

## Abstract

Background: Treatment of large articular cartilage lesions of the knee includes surgical options one of which includes cartilage replacement therapies. Among these therapies include osteochondral allograft (OCA) transplantation, which can be performed utilizing a BioUni® (Arthrex BioUni® Instrumentation System; Arthrex, Naples, FL) replacement and a ‘snowman’ technique of repair.

Hypothesis/purpose: To compare clinical and radiographic outcomes in patients who have undergone multiplug OCA transplantations utilizing a BioUni® replacement and a ‘snowman’ technique of repair.

Methods: Patients who underwent OCA transplantation utilizing a snowman technique or BioUni® replacement between January 1^st^, 2012 and December 31^st^, 2018, and who had a minimum 1-year follow-up at the same institution were identified for inclusion in this study via current procedural terminology (CPT) codes. Charts of included patients were reviewed for injury and treatment details as well as demographic information. Imaging studies and operative reports were reviewed and pre and postoperative subjective and objective outcome measures were recorded.

Results: Twenty-eight patients underwent OCA transplantation with either BioUni® replacement (n=5) or with snowman technique repair (n=23). Defects in both groups had similar characteristics including size, area, location, and classifications. Patient-reported outcomes using the Knee Injury and Osteoarthritis Outcome Score-Joint Replacement (KOOS-JR), International Knee Documentation Committee (IKDC), and Physical Health Composite Score (PCS-12) were similar at baseline and increased post-operatively for both groups with no significant differences between techniques after a mean follow-up of 2.77 ± 0.83. Although it did not reach significance, the snowman group had higher rates of knee-related complications (13%) and need for revision surgery (22%) when compared to BioUni® (0% and 0%, respectively).

Conclusion: The use of both BioUni® and snowman techniques for large, unicondylar articular cartilage lesions of the femoral condyle demonstrate improved patient-reported outcomes at short-term follow-up. The use of the snowman technique presents relatively higher rates of revision similar to previous studies with no statistical difference in patient-reported outcomes when compared to those of a single plug OCA using a BioUni® system.

## Introduction

Articular cartilage lesions in young, active patients are not uncommon, occurring in 5% of those younger than 40 years and more than 60% of all arthroscopically examined knees [1-3]. Of these, non-isolated cartilage lesions account for up to 70%, and isolated lesions account for 30% of arthroscopically examined knees [1]. Treatment of osteochondral lesions of the knee varies depending on multiple factors including location, area, and depth. Smaller chondral lesions (< 2 cm^2^) are amenable to treatment with marrow stimulation techniques while larger lesions require cartilage replacement therapy, such as osteochondral allograft (OCA) transplantation or autologous chondrocyte implantation (ACI) [4]. For those not amenable to marrow stimulation, two popular OCA cartilage replacement techniques include a BioUni® (Arthrex BioUni® Instrumentation System; Arthrex, Naples, FL) cartilage replacement and multiple overlapping plugs, the so-called “snowman” technique repair. Both options are indicated for large oval-shaped lesions that are unable to be covered by a single circular plug. The BioUni® cartilage replacement technique uses a single continuous oblong plug, whereas, the snowman technique consists of two or more circular osteochondral plugs adjacent and overlapping each other.

When utilizing a BioUni® replacement, the thickness of the underlying subchondral bone has variable lengths at different locations as opposed to a specific graft plug thickness with the snowman technique. When replacing osteochondral defects with allograft, prior studies have demonstrated incomplete allograft incorporation by creeping substitution and potential immunologic response as two factors implicated in OCA failure [5-9]. With the success of OCA relying on sufficient subchondral integration and remodeling, several authors recommend transplanting the least amount of bone marrow element containing subchondral bone to decrease the risk of adverse effects [5,9,10]. Currently, OCA plugs are recommended to be between 6 and 9 mm thick with 2 to 3 mm of cartilage and 3 to 6 mm of subchondral bone to maximize neo-vascularization and incorporation [8,9]. Prior biomechanical studies have demonstrated short plugs (< 4 mm) have significantly less pull-out strength than longer plugs of > 7 mm, and another study showed no difference between 7 mm and 10 mm plugs [10,11]. BioUni® provides approximately 10 mm of bone restoration at its maximum depth, which allows it to have significantly more bone centrally when compared to the two plugs used in the snowman configuration [12,13]. Prior studies have evaluated the outcomes of multiplug OCA transplantation (snowman technique) and demonstrated improvement in post-operative outcome measures but inferior results to single graft transplant utilizing a dowel technique [14].

To our knowledge, this is the first study to directly compare OCA replacement using a BioUni® replacement and the snowman repair technique. The authors hypothesize that there will be no significant difference in patient-reported outcomes and revision rates between these two techniques.

## Materials and methods

Patient identification

Thomas Jefferson University Institutional Review Board approval was obtained prior to the start of this study. All patients were identified by current procedural terminology (CPT) code 27415 (OCA) that underwent transplantation between January 1st, 2012 and December 31st, 2018 at a single institution. A total of 29 patients with symptomatic osteochondral lesions of the knee confirmed by arthroscopy were identified. Subjects who underwent OCA utilizing a BioUni® replacement or snowman technique with a minimum of 1-year follow-up were included. Subjects were included if their operative report, imaging, and postoperative outcome scores were available. Patients without postoperative outcomes were excluded. Patient charts from a single institution were reviewed to determine primary etiopathogenesis, pre-operative treatments, and patient demographic information.

Data collection

MRI studies were reviewed to characterize the lesion size, site, and depth and compared to intraoperative arthroscopic findings. Outerbridge classification of chondral lesions during primary arthroscopy was used to classify the severity of injury [15,16]. Operative reports of initial arthroscopy and definitive surgery were reviewed to determine lesion size, graft location, and configuration at final surgery. Postoperative subjective and objective outcome measures were recorded as well as the incidence of complications and revisions. Complications were subdivided into knee-related complications, which consisted of adverse events directly related to the graft construct or knee, and medical-related complications, which were defined as adverse events indirectly related to the operation that required medical intervention. Pre and postoperative subjective and objective outcomes were measured using the Knee Injury and Osteoarthritis Outcome Score-Joint Replacement (KOOS-JR), International Knee Documentation Committee (IKDC), Mental health Composite Score (MCS-12), and Physical Health Composite Score (PCS-12) to evaluate clinical outcomes [17,18]. The results were obtained preoperatively using these instruments and at the final follow-up. To avoid examiner bias, clinical scoring was evaluated by two independent observers (Initially Blinded for Review) who were not involved in the surgical treatment of the patients. The duration of follow-up was recorded as the date of patient response to the clinical outcome score and only patients with a minimum of 1-year follow-up were examined.

Statistical analysis

To determine the significance of intergroup differences, a 2-tailed t-test or Mann-Whitney-U test (continuous variables) and Chi-square or Fisher exact test (categorical variables) was used for age, BMI, sex, defect size, number, location, graft size, KOOS-JR, IKDC, MCS-12, and PCS-12 scores. P values < 0.05 were considered to be significant and all analyses were independently reviewed by a statistician.

Surgical techniques

In our practice, most patients with osteochondral pathology from osteochondritis dissecans are treated with fresh OCA. For those with chondral pathology only, it was at the surgeon’s discretion to perform fresh OCA as opposed to an alternative treatment option such as autologous chondrocyte implantation for the defect. The decision between BioUni® and snowman comes down to the availability of the graft and cost due to the paucity of literature on the superiority of one over the other. Overall, OCA transplantation is indicated for symptomatic younger patients with magnetic resonance imaging (MRI) evidence of focal defects measuring > 2 cm^2^ that failed to improve with conservative management or patients requiring revision procedures if there was no radiographic evidence of Kellgren-Lawrence grade 3 or 4 osteoarthritis. Full-length standing lower extremity X-rays are obtained only in patients with clinical evidence of Varus/Valgus malalignment requiring osteotomy. All knees receive preoperative MRIs, which are reviewed for the degree of pathology, lesion size, location, and involvement of subchondral bone.

Snowman Technique

The knee is draped in a standard sterile surgical fashion. Midline incision is performed, creating full-thickness skin flaps allowing medial or lateral arthrotomy. Manipulation of the patella and the knee allows exposure of chondral lesions. Once identified a pin is drilled perpendicular to the lesion and an appropriate-sized reamer is used to ream to a depth of around 8 mm. Depth measurements are taken at 4 locations at the bottom of the site. From the same or similar corresponding site, an appropriate-sized allograft plug is taken and prepared for depth measurements. Small 1 mm perforations are made in the back of the plug and the plug is pulse-lavaged to visibly remove marrow elements. Once complete, the plug is pressed to fit into place. At this point, a second perpendicular plug is drilled and once again an appropriate size gauge is used to cover the remaining lesion area. The lesion is reamed, taking out a portion of the original plug as well as the remaining lesion. Once again four different depth measurements are taken, an allograft is taken from a similar location, and measurements are applied to the allograft. Allograft is pressed fit into place and the knee is brought through the range of motion. Following copious irrigation of the surgical site, arthrotomy is closed, followed by the closure of subcutaneous tissue and skin and appropriate dressing is applied.

BioUni® Technique

The surgical approach is identical to that of the snowman technique. Once the lesion is identified, the allograft is prepared from fresh condyle, sized with the BioUni® sizer, and fixed with a guide pin. BioUni® instrumentation is then utilized to harvest a graft of exact size. The recipient site is then prepared using a sizing block, with pins placed superior and inferior. It is reamed on both sides and the slot is used to remove the edges. A trial is placed to ensure anatomic sizing. Pulsatile lavage of the graft is performed before impacting the graft into place.

**Figure 1:**
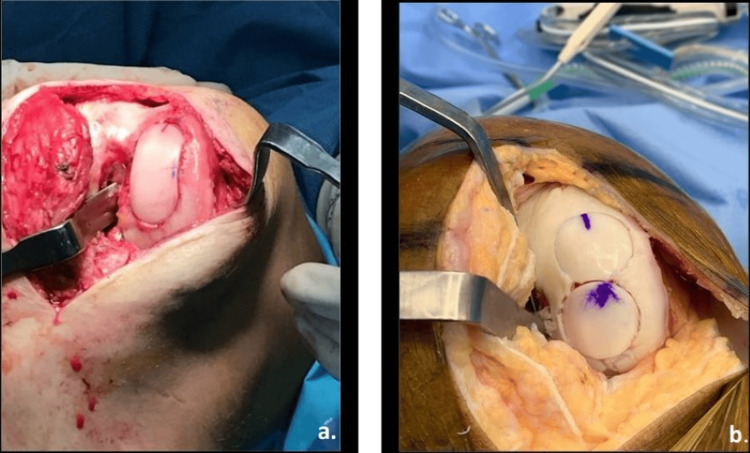
Surgical techniques (BioUni® vs snowman) (a) Intraoperative image of Arthrex BioUni® Instrumentation System (Arthrex, Naples, FL); (b) Intraoperative image of snowman technique.

Intraoperative images for both surgical techniques are shown in Figure 1.

Post-operative management

Patients are made non-weight bearing for up to six weeks following transplantation in order to protect the graft from load and shear forces. Immediate range of motion is permitted and passive and active knee range of motion is not restricted. Patients are then encouraged to undergo early strengthening and gait normalization, progressing to weight-bearing and balance exercises. Time (minimum 12 weeks from surgery) and functional recovery should both be used as criteria to return to impact activities [19].

## Results

Our cohort examined 28 patients who underwent allograft transplantation of symptomatic osteochondral lesions of the knee (Table 1). Of these, five received BioUni®, while 23 received a snowman configuration at final surgery. There was a significant difference in age between cohorts (P=.004), with the snowman group having an overall younger mean age. There were no significant differences in BMI and sex between groups. The number of defects ranged from 1-4 with greater than two defects found only in the snowman treatment group (Table 2).

**Table 1:** Patient Characteristics BMI=Body Mass Index; Bolded values indicate significance BioUni® (Arthrex BioUni® Instrumentation System; Arthrex, Naples, FL)

	Total	BioUni®	Snowman	P Value
	N=28	N=5	N=23	
Age	37.5 ± 9.01	47.4 ± 5.66	35.3 ± 8.15	0.004
BMI	27.5 ± 4.02	26.9 ± 2.99	27.6 ± 4.26	0.709
Sex:				1.000
Female	16 (57.1%)	3 (60.0%)	13 (56.5%)	
Male	12 (42.9%)	2 (40.0%)	10 (43.5%)	
Follow-up (years)	2.77 ± 0.83	2.47 ± 0.40	2.84 ± 0.89	

**Table 2:** Lesion Characteristics LFC=Lateral femoral Condyle; MFC=Medial Femoral Condyle BioUni® (Arthrex BioUni® Instrumentation System; Arthrex, Naples, FL)

	Total	BioUni®	Snowman	P Value
	N=28	N=5	N=23	
Number of Defects:				0.567
1	13 (46.4%)	4 (80.0%)	9 (39.1%)	
2	7 (25.0%)	1 (20.0%)	6 (26.1%)	
3	6 (21.4%)	0 (0.00%)	6 (26.1%)	
4	2 (7.14%)	0 (0.00%)	2 (8.70%)	
Defect Location:				0.903
LFC	12 (42.9%)	3 (60.0%)	9 (39.1%)	
MFC	4 (14.3%)	0 (0.00%)	4 (17.4%)	
patella	3 (10.7%)	0 (0.00%)	3 (13.0%)	
trochlea	9 (32.1%)	2 (40.0%)	7 (30.4%)	
Defect Area (mm^2^)	471 ±257	345 ±146	499 ±270	0.313
Outerbridge:				1.000
2	1 (3.57%)	0 (0.00%)	1 (4.35%)	
3	4 (14.3%)	1 (20.0%)	3 (13.0%)	
4	23 (82.1%)	4 (80.0%)	19 (82.6%)	

Changes in patient-reported outcome measures were found to be equivalent between treatments with no significant difference between pre and post-operative patient-reported outcomes (Table 3). Outcome scores for both the BioUni® and snowman technique increased at the final follow-up for IKDC, KOOS-JR, and PCS-12. MCS-12 scores averaged lower at the final follow-up for both cohorts.

**Table 3:** Subject Patient Reported Outcome Measurements (PROMs) Δ = indicates the change between preoperative (Pre Op) and postoperative (Post Op) PROMs.  IKDC=International Knee Documentation Committee; KOOS-JR=Knee injury and Osteoarthritis Outcome Score-Joint Replacement; PCS-12 =Physical Composite Score; MCS-12=Mental Composite Score

Patient Reported Outcome Measure	Total	BioUni®	Snowman	P Value
	N=28	N=5	N=23	
Pre OP IKDC	38.9 ±16.5	42.2 ±9.27	38.3 ±17.7	0.538
Pre Op KOOS-JR	50.7 ±13.7	51.6 ±1.42	50.5 ±15.5	0.798
Pre Op PCS-12	35.3 ±8.81	31.6 ±5.07	36.0 ±9.31	0.219
Pre Op MCS 12	54.9 ±12.6	62.4 ±5.22	53.3 ±13.2	0.215
Post OP IKDC	67.2 ±18.5	60.6 ±14.2	68.4 ±19.2	0.382
Post Op KOOS-JR	80.7 ±15.2	78.3 ±11.5	81.2 ±16.0	0.586
Post Op PCS-12	48.8 ±8.93	43.3 ±13.1	49.7 ±7.99	0.227
Post Op MCS 12	50.9 ±11.7	55.7 ±12.3	50.0 ±11.7	0.255
Δ IKDC	28.3 ±20.5	18.4 ±22.9	30.4 ±20.0	0.385
Δ KOOS-JR	27.8 ±14.6	27.6 ±6.86	27.9 ±16.2	0.970
Δ PCS-12	13.7 ±9.65	11.7 ±12.1	14.1 ±9.41	0.729
Δ MCS 12	-2.56 ±10.6	-6.71 ±10.7	-1.69 ±10.6	0.438

Complications and revisions

At a mean follow-up of 2.77 years (2.8 and 2.5 years for the BioUni® and snowman groups, respectively) there were five revisions (21.7%) in the snowman group with patients ranging from 23 to 52 years old (Table 4). Three of the five revision patients required total knee arthroplasty (13%), while the remaining two revisions consisted of a revised OCA of the patella with tibial tubercle osteotomy and open lateral lengthening and a Lateral femoral Condyle (LFC) graft failure with revision OCA. Although there were no reported revisions for the BioUni® cohort, this was not found to be statistically significant (P = 0.250).

**Table 4:** Rates of Complications and Revisions

	Total	BioUni®	Snowman	P Value
	N=28	N=5	N=23	
Medical Complications:				0.218
No	25 (89.3%)	4 (80.0%)	22 (95.7%)	
Yes	3 (10.7%)	1 (20.0%)	1 (4.3%)	
Knee-Related Complications:				0.392
No	25 (89.3%)	5 (100%)	20 (87%)	
Yes	3 (10.7%)	0 (0%)	3 (13%)	
Revision/Reoperation:				0.250
No	23 (82.1%)	5 (100%)	18 (78.2%)	
Yes	5 (17.9%)	0 (0.00%)	5 (21.7%)	

In the same follow-up period, there were a total of five reported complications. Complications were separated into two categories, medical and knee-related complications. Medical complications were defined as issues arising as a consequence of surgery that required non-surgical intervention, such as pulmonary embolism and deep vein thrombosis, while knee-related complications were defined as adverse events directly related to the graft construct or knee. Knee-related complications (13.0%, n=3) were reported only in the snowman group and consisted of knee stiffness that required manipulation under anesthesia (MUA) and two instances of graft failure to incorporate. In regard to medical complications, one patient from each group was treated medically, consisting of a pulmonary embolism (4.3%) in the snowman group and a deep vein thrombosis (DVT) (20%) in the BioUni® group. There were no reported cases of infection in either cohort. The incidence of medical and knee-related complications was not found to be statistically significant (P = 0.218 and 0.392, respectively).

## Discussion

The principle findings of this study were that, similar to the snowman technique, BioUni® OCA led to low rates of complications with both techniques resulting in improvements in the majority of postoperative functional scores that did not differ significantly between cohorts. A higher rate of reoperation was seen in the snowman group, however, it failed to reach statistical significance.

To date, there is limited published data regarding the clinical outcomes of patients treated with OCA using the dowel technique in the form of two overlapping allograft plugs for a single, elongated lesion in the knee. In biomechanical studies, the pull-out strength for oblong single-plug OCAs has been found to be significantly lower when compared to large cylindrical or multi-plug OCAs [13]. When examined in a cohort of 26 patients, Cotter et al. found that patients undergoing snowman OCA had significant clinical improvement, however, there was also a high rate of reoperation (44.4%, n=4) and a high rate of failure (33.3%, n=3) at a follow-up of 7.7 years [20]. Similarly, Cotter et al. demonstrated higher graft failure rates (33.3%, n=3) when the snowman technique was used to treat elongated cartilage lesions when compared to patients who underwent isolated, single graft transplants [14]. The results of these studies are consistent with the results of the current cohort, demonstrating that the snowman technique may result in improved clinical outcome scores for a majority of patients, although there is a relatively high revision rate when compared to a single plug OCA.

The larger graft sizes used in these techniques predispose them to collapse and fragmentation, which are the main sources of failure with rates of approximately 25% (72) at 12 years of follow-up [21,22]. Failure of OCA’s can also be attributed to the limitations of integration at the bone-to-bone interface where radiological studies have shown the development of sclerosis and subchondral cyst formation as limiting factors [23]. Traditionally, OCA transplantation was reserved for younger patients since patients older than 40 had a higher likelihood of graft failure [24]. Recent studies have shown mixed results on the effect of age on graft failure rates. Levy et al. found that patients 30 years and older had a 3.5 times higher likelihood of allograft failure [25]. A systematic review by Kunze et al. examined the influence of age on graft failure and demonstrated that older age at the time of surgery was significantly associated with failure after OCA among the studies that treated age as a continuous variable [26]. The predilection towards worse outcomes in older patients is presumed by many to be due to pre-existing joint degeneration. Markus et al. [27] further examined this notion by analyzing a cohort of patients > 45 years old that excluded patients with a history of knee osteoarthritis or inflammatory joint disease and found a graft survival rate of 100% (18) at 3.1 years [27]. While there is conflicting evidence in the literature, studies have shown that patients ≥40 years old undergoing OCA transplantation can delay the need for joint replacements and improve their quality of life [25,27-30]. In the current study, the significant difference in age between our cohorts did not reveal itself in the form of worse clinical outcomes, although it was not explicitly analyzed here.

Limitations

This study had several limitations. Firstly, the limited number of patients that were eligible for inclusion, particularly in the BioUni® group and thus places this study at high risk for Type II error. Secondly, there was a lack of long-term follow-up data, and although OCA is a good midterm surgical option, longer-term follow-up studies are needed to further elucidate differences in clinical outcomes amongst surgical techniques. Despite these limitations, the authors feel that the study captures a clinically relevant analysis of outcomes in two commonly used surgical techniques. Future high-quality prospective studies in larger cohorts should be completed to further evaluate these techniques.

## Conclusions

In conclusion, this study demonstrates that both the BioUni® and snowman techniques for large, unicondylar articular cartilage lesions have improved patient-reported outcomes at short-term follow-up. However, patient-reported outcomes do not significantly differ between both techniques. Furthermore, the snowman technique presented higher rates of revision when compared to the BioUni® system.
